# The Global Perspective of Nursing Students in Relation to College Peers

**DOI:** 10.5539/gjhs.v7n2p235

**Published:** 2014-10-28

**Authors:** Eman Allam, Mary E. Riner

**Affiliations:** 1 Indiana University Purdue University Indianapolis, Indiana, USA; 2 National Research Centre, Cairo, Egypt

**Keywords:** global perspective, nursing education, cultural competence

## Abstract

**Objective:**

The purpose of the present study was to assess the nursing students’ global perspectives and compare it to a nationally normed reference group.

**Background:**

An individual’s global perspective impacts the extent to which the person perceives and knows the people and cultures within the world. Nursing care is expected to take a holistic perspective in providing care and respond in culturally appropriate ways to a diverse population through understanding the impact of cultural influences.

**Methods:**

Participant nursing students completed the Global Perspective Inventory survey and information about their current global perspective taking and their perceptions of curricular and co-curricular experiences was collected and analyzed.

**Results:**

Compared to the nationally normed reference group, nursing students expressed statistically significant lower intrapersonal affect average score. Although higher average scores were detected in most of the other scales, differences were not statistically significant.

**Conclusion:**

A global perspective approach to intercultural nursing education is an area that needs to be further developed and different options are to be examined.

## 1. Introduction

A rapidly changing and increasingly global, multicultural world requires complex skills and knowledge from citizens. As a knowledge-based profession, nursing schools must be capable of developing within students the necessary capabilities to face the challenges of emerging global conditions, together with an awareness of shared values and belonging to a common social and cultural space. The importance of education in the development and strengthening of stable, peaceful and democratic societies is universally acknowledged as paramount.

The value of including cultural competence and global perspective taking in nursing education can be thought of from three philosophical positions (Stier, 2006). The first, idealism, derives from the assumption that internationalization is good per se and serves to highlight global life-conditions and social injustices and offers an emancipatory worldview. The second, instrumentalism considers internationalization as a viable road to meeting healthcare organizations needs for nursing staff to meet demands of diverse patients for high quality care. The third, educationalism, recognizes the personal or societal value of learning itself. Exposure to ‘strange’ cultures with its features, social expectations and language-requirements is considered a unique multilevel learning experience where intercultural competence, knowledge of and respect for other cultures may be developed.

The current global nature of our society resulted in an increased interest in global education in academia. Implementing global education within college curricula is expected to prepare students to function as global citizens in a diverse world where technology continues to bring people together from all over the world. It also increases students’ opportunities to pursue careers in international settings. Undergraduate health professions in specific have increasingly sought to incorporate a global perspective into the curriculums in order to enhance the students’ total college experience and prepare them as global citizens seeking to improve health of populations abroad (Green & Olson, 2003; Gacel-Avila, 2005; Dutschke, 2009).

Evidence exists that study abroad experiences, for example, have a prevailing impact on students’ attitudes, intercultural skills and learning within a discipline. They offer students the chance to learn about global diversity and the interrelationships of local, national, and international issues affecting the world’s population today. Study abroad programs are considered an integral component of effective global education. They provide students with opportunities to learn formally and informally outside of the institution, the native country, and the learners’ comfort zone (Bolen, 2007). These experiences are also significantly important for attaining institutional internationalization goals (Braskamp, et al., 2009).

With the increasing importance of global education and global perspective-taking comes an increased need for a way of measuring attainment of skills, knowledge and attitudes. The Global Perspective Inventory (GPI) was introduced to the research community for this purpose. The GPI is a survey instrument that measures participants’ global perspective in terms of cognitive, intrapersonal, and interpersonal domains - each in terms of both development and acquisition (Merrill et al., 2012). It was proposed that this inventory is a holistic approach to the measurement of global perspective-taking because, unlike other scales, it takes into account intercultural maturity and communication (Merrill et al., 2012).

After establishing an office for global affairs in a public research university, the advisory committee decided to conduct an assessment of current students’ global perspectives. This was intended to be a baseline assessment that could be used to guide future programming and curriculum work to further the process of internationalizing the curriculum. We also wanted to benchmark our nursing students with a nationally normed reference group. The GPI was used to measure the nursing students’ perception of the campus environment and their global perspectives.

## 2. Literature Review

An individual’s global perspective impacts the extent to which the person perceives and knows the people and cultures within the world. It includes an individuals’ sense of people, nation, and world beyond themselves. Colleges and universities have instituted and refined initiatives such as study abroad, diversity education, and multicultural curricula with the intention of developing students’ global perspectives, but little evidence regarding their effectiveness exists (Musil, 2006).

Nursing care within the treatment model, in specific, emphasizes providing care within a diagnostic and treatment regime for patients, with little consideration of a cultural perspective. However, nurses are expected to take a holistic perspective in providing care and respond in culturally appropriate ways to a diverse range of individuals through understanding the impact of a range of culturally significant factors on the health and healthcare of the individual. Developments in the field of transcultural nursing have contributed to curriculum guidelines, and a number of theoretical perspectives and approaches have emerged regarding the curriculum content and the most appropriate way of teaching transcultural care (Lynam, 1992; Leininger, 1994; Andrews, 1995).

The fact that all nursing students must be given the opportunity to apply the theoretical knowledge and skills acquired to meet the healthcare needs of those from different cultures in practice was recognized by Lynam (1992). Similarly, Bartz et al. (1993) considered that students must be given the opportunity to practice transcultural nursing and not simply be taught the theory underpinning cultural assessment. The literature identifies various possible options for gaining experience with different cultural/ethnic groups, amongst which is the inclusion of an international experience for students undertaking their initial nurse training program.

The long-term effects of study abroad experiences on American nursing students were investigated by Zorn (1996). She studied the impact of the international experience in relation to four dimensions (professional career, intellectual development, global understanding and personal development). Zorn reported the highest impact in enhanced international perspective and personal development with a lower impact reported in the professional nurse role and particularly in intellectual development. The study abroad programs varied in their duration and students who had participated in longer programs reported a greater impact than those participating in shorter experiences. The length of time that had elapsed since the international program showed a negative correlation with the degree of impact but the longer experience resulted in a greater long-term impact.

Zhai and Sheer (2004) found that of a sample of 145 undergraduate, those students who had more contact with people from other countries had higher levels of global perspective scores. Student attitudes toward cultural diversity were also found to be more positive. In this study, females were found to be more positive toward cultural diversity than males. Their results also showed that there were a high correlation between a student’s grade point average and his/her attitudes toward diversity.

According to Thompson et al. (2000), the need for nursing students to have a full mindfulness of the influence of cultural factors on health and healthcare is central if they are to respond appropriately to the cultural diversity that exists in society. They stated that understanding the influence of culture on health, awareness of the impact of one’s own ethnic background on interaction with others and sensitivity towards the ethnicity of others should be central concepts in nursing curricula.

Kelleher (2005) created a global learning assessment matrix that included a four-phase education continuum which underscores the various dimensions of an integrative and developmental global learning process for students. Musil (2006) developed a global learning assessment framework that included goals, outcomes and assessment strategies. The goals included generating new knowledge about global studies, spurring greater civic engagement and social responsibility, promoting deeper knowledge and debate about the practice of democracy, and cultivating intercultural competencies. This global learning assessment matrix represents a framework to guide campus assessment in regards to improvement of the students’ global learning in future courses.

Glass et al. (2013) confirmed the existence of disturbing trends reported in major higher education periodicals regarding the globally mobile students’ experiences and indicated that the main reasons are the lack of sense of community, low quality faculty-student interactions, and the uneven global learning. They stated that the social and psychological influences are the key environmental factors that shape the students’ global perspective development. They concluded that richer and “higher-diversity” courses enable students to connect more with other students, faculty, and the institution at large and are necessary for fostering positive student global learning and development.

Chickering and Braskamp (2009) indicated that it is essential that the traditional-aged college students develop and internalize a global perspective into their thinking, sense of identity, and relationships with others. They argued that, given the changing societal demands and expectations, the holistic student developmental perspective could be enhanced through four “vectors” that were identified and defined as important in the psychosocial development of college students. These four vectors are: moving through autonomy toward interdependence, establishing identity, developing purpose, and managing emotions.

Although the impact of global education experiences are still not well documented and often based on assumptions made by the educator, the increase in study abroad and international programs enrollment should lead governments, institutions, and faculty to focus on the effectiveness of teaching and learning in these programs (Dekaney, 2008, Vande Berg 2007). If global education experiences are to facilitate learning effectively, faculty in these programs must intervene before, during, and after these experiences. Green (2007) also contends that faculty must develop an internationalized and global mindset to create learning that is comparative, integrative, interdisciplinary, and contextual.

## 3. Methods

Approval to conduct this study was obtained from the university *Institutional Review Board* committee. The study population consisted of all the School of Nursing Bachelor of Science students, including both first and second degree students, enrolled in the spring 2013 semester. To recruit the students, a flyer was displayed on the kiosk on the first floor of the school and an informational email was sent to all students. One week after, another email was sent with an invitation to participate in the study including the link to the survey and the access code to take the survey. Students were asked to complete the survey within three weeks. A reminder email was sent out approximately two weeks after the initial invitation. Then, a second reminder was sent three days later.

The Global Perspective Inventory (GPI) (Braskamp, Braskamp, Merrill, & Engberg, 2012) was used as the measurement tool. This web-based survey instrument collects information from students about their current global perspective taking and their perceptions of curricular and co-curricular experiences. Student responses are combined into six scale scores that summarize three dimensions of global perspective taking (cognitive, intrapersonal, and interpersonal) with two different scales for each dimension. Students also answer questions about the frequency and quality of global learning opportunities and experiences in several curricular and co-curricular activities, classified by curriculum, co-curriculum, and community (See Appendix A for a definition of the global perspective taking scales and Appendix B for list of items included in the survey).

Developed by Braskamp, Braskamp, Merrill, and Engberg (2010) the GPI includes eighty-one-items that measure students’ development along each of the cognitive, intrapersonal, and interpersonal domains as well as their engagement with the social and academic environment of their colleges. The fundamental questions of the GPI translate into the six empirically validated scales that are related to the global perspective research question.

The two cognitive scales are designed to assess *knowing* (degree of complexity of one’s view of the importance of cultural context in judging what is important) and *knowledge* (degree of understanding and awareness of various cultures). Thus, the first scale focuses on how one approaches thinking and learning, whereas the second scale reflects what one knows and understands about our global world. The intrapersonal scales measure aspects of *identity* (level of awareness of one’s unique identity and sense of purpose) and *affect* (level of respect for and acceptance of cultural perspectives different from one’s own and degree of emotional confidence when living in complex situations). The interpersonal scales capture elements of *social responsibility* (level of interdependence and social concern for others) and *social interaction* (degree of engagement with others who are different from oneself and degree of cultural sensitivity in living in pluralistic settings) (Merrill et al., 2012).

The GPI as an overall tool was reported to be reliable and alphas for the subscales ranged from 0.65 to 0.77 (Merrill et al., 2012). Students responded to the 81 global perspective taking items using a strongly agree (SA) to strongly disagree (SD) continuum. Scale scores were then calculated for each of the six scales. Results were then compared to the “norm average”. The norm average represents the average response of each scale and item of all undergraduate students attending private and public four years institutions who completed the questionnaire the past three academic years (Braskamp, 2014). This sample of undergraduates that was included in the national norm groups was selected from a larger group of approximately 5100 students who completed the GPI from August 2010 to May 2013. Of the selected undergarduate students, 44.2% were enrolled in private colleges offering bachelors and graduate degrees. About 35.5% were enrolled in public universities. Approximately 95% of these students were traditional aged (Braskamp, 2014). Comparison was done using t-test and statistical significance was assumed at p < 0.05.

## 4. Results

The sample included 127 nursing students with mean age of 26 ±5.48 years. This represents a 13% response rate for the nursing student body. About 89% of the survey respondents were females with 86% identifying themselves as white with the others divided among the other ethnic categories. The students’ demographic characteristics are presented in Table 1.

**Table 1:** Demographic characteristics of the participant students

Gender	Percent
Male	9.8%
Female	89.4%
Other	0.8%

**Ethnic Identity**	

Mutiple Ethnities	0.8%
African	5.7%
Asian	0.8%
European	86.2%
Hispanic/Latino	4.1%
Native American	0.0%
I prefer not to respond	2.4%

**School Status**	

Sophomore	17.9%
Junior	33.3%
Senior	41.5%
Graduate Student	0.0%
Other	6.5%
Faculty	0.8%

**College GPA**	

A+ or A	41.5%
A-	35.0%
B+	16.3%
B	7.3%
C	0.0%
D	0.0%

**Parent Highest Education**	

High school or less	11.4%
College degree	67.5%
Graduate degree	21.2%

**Study Abroad**	

No Terms	80.5%
Short Term Only	12.2%
One Term	2.4%
Two Terms	1.6%
More than Two Terms	3.3%

**Participated in Living Learning Program**	

Yes	14.6%
No	85.4%

**Figure 1:**
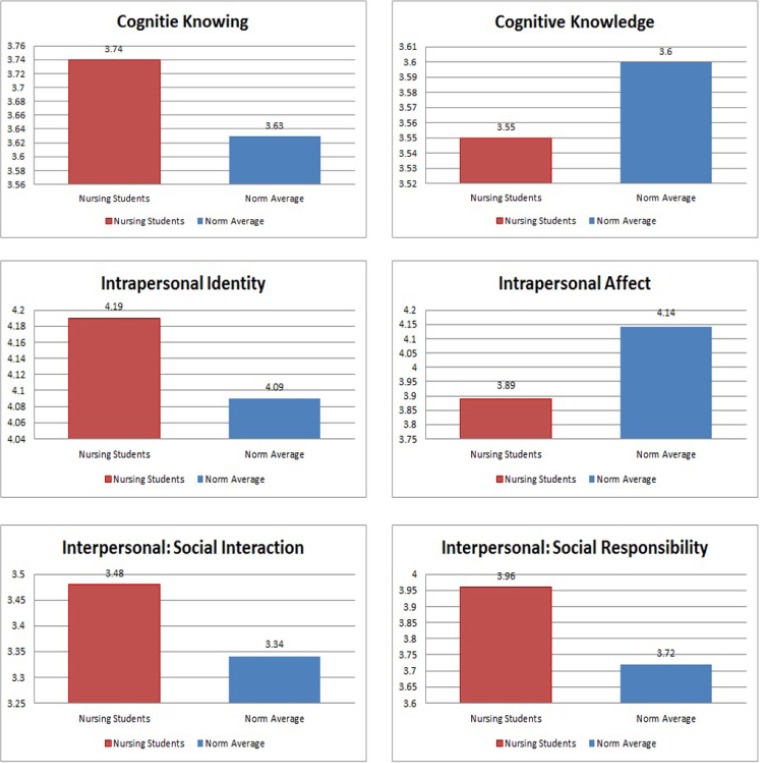
Comparisons between nursing group means and the norm referenced group means for all GPI scales

The average score of each scale and the comparison between the two groups are presented in Figure 1 and Table 2. Although higher average scores were detected in most of the scales for the nursing students, differences were not statistically significant. Nursing students expressed statistically significant lower intrapersonal affect average score.

**Table 2:** Mean scores of the GPI

	Cognitie Knowing	Cognitive Knowledge	Intrapersonal Identity	Intrapersonal Affect	Interpersonal: Social Interaction	Interpersonal: Social Responsibility
Nursing group	3.74	3.55	4.19	3.89	3.48	3.96
Norm Average	3.63	3.60	4.09	4.14	3.34	3.72
*p*-value	0.10	0.45	0.08	0.03*	0.10	0.59

* p<.05.

## 5. Discussion

The GPI reflects a global and holistic view of the students’ learning and development and the importance of the campus environment in fostering holistic student development. It measures how a student thinks, views him/herself as a person with a cultural heritage, and relates to others from other cultures, backgrounds and values. The present study compared the global perspectives of the nursing students with national norms of students from multiple disciplines.

Nursing educators all over the world are gradually recognizing the importance of exposing students to a range of learning experiences that will broaden their global perspective. These experiences can generally occur through enhancing the international exchanges, study abroad programs, university partnerships, and globally-focused curriculum changes (Bolan, 2003).

Nurses are responsible for applying the basics of research and clinical practice to influence the health of the population. A global health perspective aims to endorse the wellbeing and equity for people globally through collaboration of many disciplines and by focus on population based prevention programs and individual clinical care (Koplan et al., 2009). Evolving cultural, economic, political, and social forces in the global community greatly affect both local and global health, nursing, and healthcare outcomes in general.

In the current study, we chose to use the Global Perspective Inventory (GPI) because it aims to measure an individual’s growth and development as a consequence of life experiences (Braskamp et al., 2010). The GPI has been used broadly with college students. It is constructed to measure how the students gain insight into the world around them, and how these insights influence their self-perception and interpersonal relationships. As a consequence, the GPI’s questions are designed to reflect the integration of its scales with daily life.

A sample of 127 (about 13% response rate) students took part in the study. Participants were selected using purposive, convenience sampling. Future research is planned with a larger study sample to overcome the sample limitations of the current study. The results of the comparison demonstrated that, although non-statistically significant, nursing students’ average score of cognitive knowing, intrapersonal identity, social interaction, and social responsibility scales were higher than national norm average. Nursing students also expressed statistically significant lower intrapersonal affect average score. These results indicate that nursing students’ level of cultural awareness and knowledge as well as their social skills are very comparable to their peers.

While the nursing students’ lower intrapersonal score was a surprise, it indicates a need for further learning experiences to develop global perspectives. From a curriculum development perspective, learning experiences are planned that will provide students opportunities to develop emotional skills. These will likely include identifying personal values and beliefs, understanding why feelings occur and their implications, developing strategies to cope with diverse feelings (e.g., xenophobia, uneasiness, uncertainty, ambiguity, frustration, anger, ethnocentrism) triggered by unknown cultural settings, and preventing them from automatically determining one’s actions or interpretations of behavior or events (Stier, 2006).

Intrapersonal competence has much in common with emotional intelligence which has been defined as including an array of noncognitive abilities, capabilities, and skills that influence one’s capacity to succeed and cope with environmental demands and pressures (Bar-On, 1997). Curriculum revisions designed to augment nursing students’ cognitive abilities can improve emotional intelligence abilities to perceive, understand, and regulate emotions (Jaeger, 2003).

Instructors need time, training, experience and willingness to help student acquire the competencies they will need to relate to patients, colleagues, and others in healthcare environments from cultures different than their own (Hoberman & Mailick 1994). Learning experiences in nursing curricula need to be designed to help students discover and improve their levels of emotional intelligence. This type of intelligence is highly valued in settings where interpersonal interactions are critical to successful delivery of care and increasingly with outcomes.

In conclusion, the findings of the present study indicate the need for nursing faculty to engage students in learning experiences that increases their intrapersonal competence with diverse others. A global perspective approach to intercultural learning is an area that needs to be further developed and different options are to be examined. Students’ global competence will greatly impact their career development in the future. It is expected to provide them with great critical thinking skills that are useful in caring for diverse patients. Nursing schools and programs are encouraged to focus on integrating global competence contents into their curriculum and offer students opportunities to enhance their transcultural care knowledge and skills.
